# The Impact of Postoperative Urinary Diversion on Surgical Outcomes of Hypospadias Repair: A Systematic Review and Meta-Analysis of Pediatric Literature

**DOI:** 10.3390/medicina61091659

**Published:** 2025-09-12

**Authors:** Maria Escolino, Maria Sofia Caracò, Valerio Mazzone, Claudia Di Mento, Francesca Carraturo, Flavio Perricone, Giovanni Esposito, Mauro Porcaro, Ciro Esposito

**Affiliations:** 1 Division of Pediatric Surgery, Federico II University Hospital, 80131 Naples, Italy; 2 CEINGE Advanced Biotechnologies, 80131 Naples, Italy; 3 Division of Pediatric Radiology, Federico II University Hospital, 80131 Naples, Italy

**Keywords:** hypospadias, urinary diversion, catheter, stent, complications, urethroplasty

## Abstract

*Background and Objectives*: The optimal postoperative urinary diversion strategy after hypospadias repair remains debated, with variability in type, material, and duration. Pediatric-specific data are limited. This systematic review and meta-analysis aimed to evaluate how urinary diversion type and duration affected surgical outcomes after pediatric hypospadias repair. *Materials and Methods*: A systematic search of PubMed, Embase and Cochrane Library (1995–2025) databases was conducted using PRISMA guidelines and registered in PROSPERO (CRD420251121638). Eligible studies included pediatric patients undergoing hypospadias repair with or without urinary diversion. Primary endpoints were diversion characteristics (type, material, duration); secondary endpoints included postoperative complications and re-operations. *Results*: A total of 31 studies representing 4261 pediatric patients were analyzed, with 16 included in meta-analysis. Pooled estimates confirmed that bladder catheters were associated with significantly higher rates of general complications (wound/urinary tract infections, swelling, foreskin dehiscence) [*p* < 0.05] and re-operations [*p* < 0.05] compared to urethral stents. Conversely, urethral stents showed a greater risk of mechanical complications such as blockage and dislodgement [*p* = 0.001]. Meta-analysis of stented versus unstented procedures revealed no significant differences in major outcomes (fistula, stenosis, re-operations), although stented repairs showed a modest increase in minor complications. For catheterization length, pooled data suggested no significant advantage of prolonged (>5 days) versus shorter (≤5 days) diversion, despite descriptive analyses indicating more functional and general complications [*p* < 0.05] and re-operations [*p* = 0.004] after short diversion and more fistulas after prolonged diversion [*p* < 0.05]. *Conclusions*: Urinary diversion strategies significantly affected surgical outcomes after pediatric hypospadias repair. Urethral stents reduced general complications compared with bladder catheters but were prone to mechanical issues. Short diversion increased early complications, while prolonged catheterization increased fistula risk. No single approach proved universally superior; therefore, perioperative management should be individualized according to patient characteristics, hypospadias severity, and intraoperative findings. Further high-quality prospective trials are needed to define optimal diversion protocols.

## 1. Introduction

Hypospadias is one of the most frequent congenital anomalies in boys, with an estimated incidence of 1 in 200–300 live births, and its surgical correction represents one of the most common procedures in pediatric urology [1,2,3]. Despite improvement in surgical techniques and perioperative care, postoperative complications such as urethrocutaneous fistula, urethral stenosis, and wound disruption remain frequent, with relevant impacts on functional and cosmetic outcomes [4]. Among the modifiable perioperative factors potentially influencing the outcomes, the use, type, and duration of urinary diversion have been among the most studied aspects [5,6].

Urinary diversion is intended to protect the neourethra, ensure urinary drainage, and reduce the risk of early postoperative complications, but its actual benefit remains debated. Bladder catheters and urethral stents are the most frequently used devices; however, both modalities are associated with potential adverse events. Bladder catheters may cause discomfort, bladder spasms, tube blockage or urinary tract infections, while urethral stents may dislodge or remove during voiding or accidentally cause urethral trauma.

Furthermore, the necessity of any urinary diversion after distal hypospadias repair has also been questioned, with some studies showing no significant difference between stented and unstented procedures [7]. Current EAU/ESPU Paediatric Urology Guidelines report that urine drainage after hypospadias repair may be achieved transurethrally (dripping stent) or via a suprapubic tube, and no drainage is also acceptable after distal repairs. They do not recommend a single diversion strategy or the optimal duration of stenting and emphasize individualized decision-making [8].

Overall, evidence remains fragmented, and pediatric-specific data on catheter type, size, and removal strategies are limited. Given this variability and the lack of high-quality evidence, clarifying the impact of urinary diversion type and duration on surgical outcomes is highly relevant for everyday clinical practice.

This systematic review and meta-analysis aimed to evaluate the influence of urinary diversion strategies, including type and duration, on postoperative outcomes after pediatric hypospadias repair and to provide evidence-based guidance for clinical decision-making.

## 2. Materials and Methods

This systematic review was conducted in accordance with the Preferred Reporting Items for Systematic Reviews and Meta-analyses (PRISMA) guidelines. This review was registered on PROSPERO (CRD420251121638) (http://www.crd.york.ac.uk/PROSPERO/, accessed on 7 August 2025).

### 2.1. Search Strategy

A comprehensive electronic literature search was conducted across PubMed, Embase and Cochrane Library databases to identify studies reporting on urinary diversion techniques used in hypospadias surgery in the pediatric population. The search strategy employed the following keywords consistent with MeSH terms: (urinary diversion OR bladder catheter OR urethral stent) AND (hypospadias OR urethroplasty OR urethral repair OR urethrocutaneous fistula OR complications) AND (children OR pediatrics). The search covered a 30-year period (1995–2025). References of relevant studies were manually screened to identify additional eligible records, and duplicates were removed.

**Figure 1:**
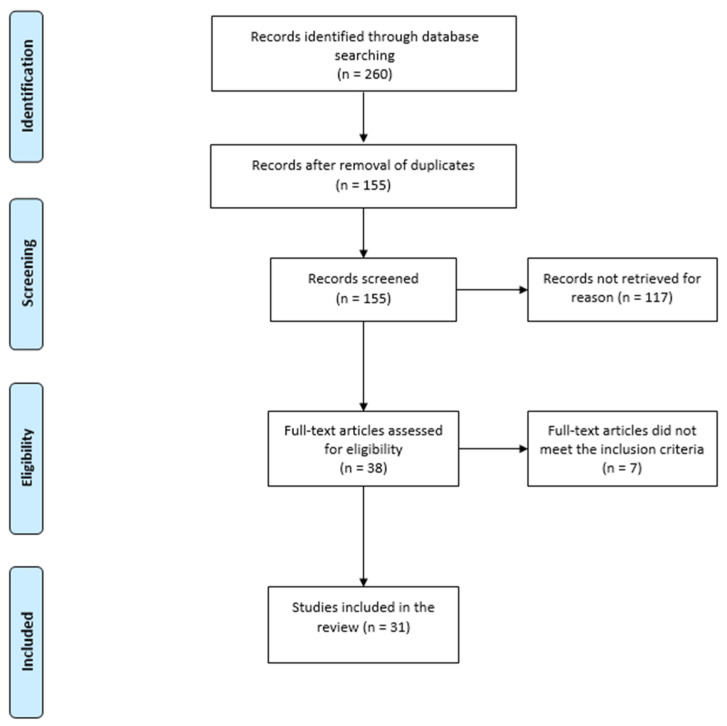
Preferred Reporting Items for Systematic Review and Meta-analysis (PRISMA) flow diagram for study selection.

A detailed PRISMA flow diagram of the search strategy is presented in Figure 1.

### 2.2. Study Selection

Inclusion and exclusion criteria were defined according to the PICO framework:

- Patient/Problem: Pediatric patients diagnosed with hypospadias (age ≤ 18 years).
- Intervention: Hypospadias repair with urinary diversion (bladder catheter or urethral stent).
- Comparison/Control: Hypospadias repair without urinary diversion (unstented procedures).
- Outcome(s): Urethrocutaneous fistula/dehiscence rate, meatal/urethral stenosis rate, rate of other postoperative complications, diversion-related mechanical and functional complications, and re-operation rates.

Eligible studies included randomized controlled trials (RCTs), prospective cohort studies, and retrospective studies reporting the type and duration of urinary diversion and postoperative outcomes. Comparative studies reporting the outcome of stented vs. unstented hypospadias repair were also included in the analysis. Only studies published in English were considered.

Exclusion criteria were studies with unavailable full texts, non-human clinical trials, and studies with incomplete outcome data or not available in English. Conference papers, case reports, reviews, and non-peer-reviewed articles were also excluded.

Study selection was performed independently by three reviewers (MSC, VM, and ME). Titles, abstracts, and full texts were screened in duplicate. Any discrepancies were resolved by discussion or, if consensus was not reached, consultation with a senior author (CE). Inter-rater reliability for the initial abstract screening phase was assessed using Cohen’s kappa statistics, which demonstrated substantial agreement (*κ* = 0.82).

### 2.3. Data Extraction

Data were extracted using a pre-defined Excel spreadsheet (Microsoft Excel, version 15.4) and included author details, study design, patient characteristics (sample size, age, degree of hypospadias, operative technique), urinary diversion characteristics (type, material, retention time), and reported outcomes (complications, re-operations). Median values with interquartile ranges were extracted for continuous variables, where applicable.

Data extraction was performed by three independent reviewers (MSC, VM, and ME) using the standardized form to ensure consistency. Discrepancies in data entry were discussed and resolved by consensus, with arbitration by a senior author (CE) when necessary. Inter-rater reliability for data extraction was also assessed, showing high agreement (*κ* = 0.85).

### 2.4. Study Endpoints

Primary endpoints included all urinary diversion-related variables, such as type, material, and duration of diversion. Secondary endpoints included postoperative complications such as urethrocutaneous fistula (UCF), glans dehiscence, meatal/urethral stenosis, mechanical complications (blockage, obstruction, dislodgement of the catheter/stent), functional complications (bladder spasm, urinary extravasation, pain or straining during voiding, urinary retention), other complications (wound infection, urinary tract infection, foreskin dehiscence) and need for re-operation.

The review was designed to address the following key questions: (1) Which type of urinary diversion is the most effective? (2) Which material should be used? (3) What is the optimal duration of diversion? (4) Is urinary diversion necessary after hypospadias repair?

### 2.5. Risk of Bias Assessment

The quality of the studies included was assessed using the Newcastle–Ottawa Scale (NOS). Three independent reviewers (MSC, VM, and ME) evaluated the studies and resolved discrepancies through discussion. A predefined data extraction form was used to ensure consistency. Interobserver agreement was high across all domains. NOS results are summarized in Supplementary Table S1, reporting individual study scores across selection, comparability, and outcome domains.

### 2.6. Statistical Analysis

Statistical analysis was conducted using Review Manager (RevMan) software, version 5.4. Odds ratios (ORs) with 95% confidence intervals (CIs) were calculated for dichotomous variables, and mean differences were reported for continuous variables. Fisher’s exact test was used to compare categorical variables. A *p* value ≤ 0.05 was considered statistically significant.

The degree of statistical heterogeneity across the included studies was assessed using the I2 statistic. A random-effects model was applied to account for expected variability across studies. In addition, publication bias was explored by visual inspection of funnel plots and formally tested with Egger’s regression asymmetry test when ≥3 comparative studies were available. A *p*-value < 0.05 was considered indicative of small-study effects.

This study was exempt from Institutional Review Board (IRB) approval, as it was a systematic review of previously published data.

## 3. Results

### 3.1. Study and Patient Characteristics

A total of 260 articles were retrieved from the initial literature search. After removal of duplicates and irrelevant studies, 155 abstracts were screened. Following the screening, 117 studies were removed based on exclusion criteria. We then reviewed the 38 remaining studies, excluding a further 7 due to unavailable full text. Finally, 31 studies were found relevant and included in the review [9,10,11,12,13,14,15,16,17,18,19,20,21,22,23,24,25,26,27,28,29,30,31,32,33,34,35,36,37,38,39].

The review included fourteen retrospective observational studies, three retrospective cohort studies, two retrospective comparative studies, three prospective observational studies, three prospective randomized clinical trials (RCTs), two prospective cohort studies, two prospective non-RCTs, one prospective cross-sectional study, and one prospective comparative study.

A total of 4261 male patients undergoing hypospadias surgery were recruited in the selected studies. The median age at time of hypospadias repair was 32.59 months (range 7–73.2). The distribution of patients according to the hypospadias degree, type of operative technique and type of postoperative urinary diversion is summarized in Supplementary Table S2.

### 3.2. Urinary Diversion Types

The included studies described various types of urinary diversions that varied in terms of material composition, duration, and specific clinical use. They were broadly categorized into two main groups: urethral stents and bladder catheters. Urethral stents were typically confined to the reconstructed urethral segment and did not extend into the bladder. Stents used for urinary drainage following hypospadias repair included the double pigtail stent [17], the Zaontz urethral stent [24], and the Koyle urethral stent [28]. All were generally secured to the glans using a suture. Conversely, bladder catheters were typically placed from the external urethral meatus into the bladder and included devices such as feeding tubes or Foley catheters.

Regarding material composition, twelve studies [11,17,20,22,23,24,27,28,35,37,38,39] reported the types of materials used, including silicone, latex, polyvinyl chloride, C-flex, and silastic. However, none of the studies clearly demonstrated a significant correlation between the diversion material and clinical outcomes.

The duration of urinary diversion varied largely among the studies. To enhance the comparability of results, we classified the studies into two subgroups based on the catheterization length: ≤5 days and >5 days.

### 3.3. Postoperative Outcomes

Urethroplasty-specific complications were described in all studies, with an overall median rate of 13.5%. They included urethrocutaneous fistula (UCF)/dehiscence (451/4261, 10.6%) and meatal/urethral stenosis (126/4261, 2.9%). Other postoperative complications, such as foreskin dehiscence, secondary phimosis, urinary tract infection (UTI), fever, wound infection (WI), hematoma, and penile swelling, were reported in 96/4261 (2.2%) subjects. Diversion-related complications, including catheter blockage, obstruction, or dislodgement, were reported in 27/4261 (0.6%). Functional complications, such as dysuria, bladder spasms, acute urinary retention (AUR), and urinary extravasation, were recorded in 167/4261 (3.9%). Finally, re-operations were reported in 21 studies, with an overall rate of 4.10%. They included fistula closure, urethral dilation, revision of penile skin, and redo-urethroplasty.

As detailed in Supplementary Table S3, the comparison between types of urinary diversion (bladder catheter vs. urethral stent) highlighted important differences. Bladder catheters were associated with higher rates of general postoperative complications, including WI, UTI, penile swelling, and foreskin dehiscence (3.3% vs. 0.8%) [OR 4.21; 95% CI 2.03–8.74; *p* < 0.05], as well as a higher re-operation rate (6.8% vs. 1.4%) [OR 5.08; 95% CI 2.95–8.73; *p* < 0.05]. In contrast, urethral stents showed a higher risk of mechanical issues, such as blockage or dislodgement (2.2% vs. 0.7%) [OR 0.30; 95% CI 0.16–0.66; *p* = 0.001]. Interestingly, the incidence of UCF or dehiscence (12.8% vs. 10.3%) and functional complications (6.2% vs. 4.6%) was slightly higher in the stent group, though not statistically significant (*p* = 0.07). Meatal/urethral stenosis occurred at similar rates in both groups (2.2% vs. 2.9%; *p* = 0.27).

When comparing stented versus unstented procedures (Supplementary Table S4), outcomes were largely comparable. The rates of UCF (7.8% vs. 6.4%, *p* = 0.27) and meatal stenosis (3.7% vs. 3.3%, *p* = 0.65) showed no significant differences. Stented repairs were associated with a slightly higher rate of other complications (6.0% vs. 4.4%, *p* = 0.16) and functional issues (9.2% vs. 6.9%, *p* = 0.09), but these differences were not statistically significant. Mechanical complications were rare but occurred almost exclusively in the stented group (0.5%) [OR 11.1; 95% CI 0.56–222.02; *p* = 0.05]. Re-operation rates were modest and similar in both groups (6.7% vs. 5.4%, *p* = 0.3). These findings suggested that the protective role of stents in maintaining urethral patency was counterbalanced by the increased risk of minor complications and mechanical issues, whereas unstented repairs did not appear to increase the risk of major postoperative problems.

The analysis of catheterization length (Supplementary Table S5) highlighted a trade-off between shorter and longer diversion times. Early catheter removal (≤5 days) was associated with significantly higher rates of functional complications (13.8% vs. 1.7%) [OR 9.13; 95% CI 5.88–14.18; *p* < 0.05], general postoperative complications (4.5% vs. 1.8%) [OR 2.62; 95% CI 1.45–4.74; *p* < 0.05], and re-operations (6.2% vs. 3.2%) [OR 2.02; 95% CI 1.23–3.3; *p* = 0.004]. Conversely, prolonged catheterization (>5 days) was associated with a higher incidence of UCF or dehiscence (14.7% vs. 5.9%) [OR 0.36; 95% CI 0.23–0.57; *p* < 0.05]. The rates of meatal stenosis did not differ significantly (3.5% vs. 4.2%; *p* = 0.52), while mechanical complications remained uncommon in both groups (≤1%).

### 3.4. Meta-Analysis of Outcomes

#### 3.4.1. Bladder Catheter vs. Urethral Stent

**Figure 2:**
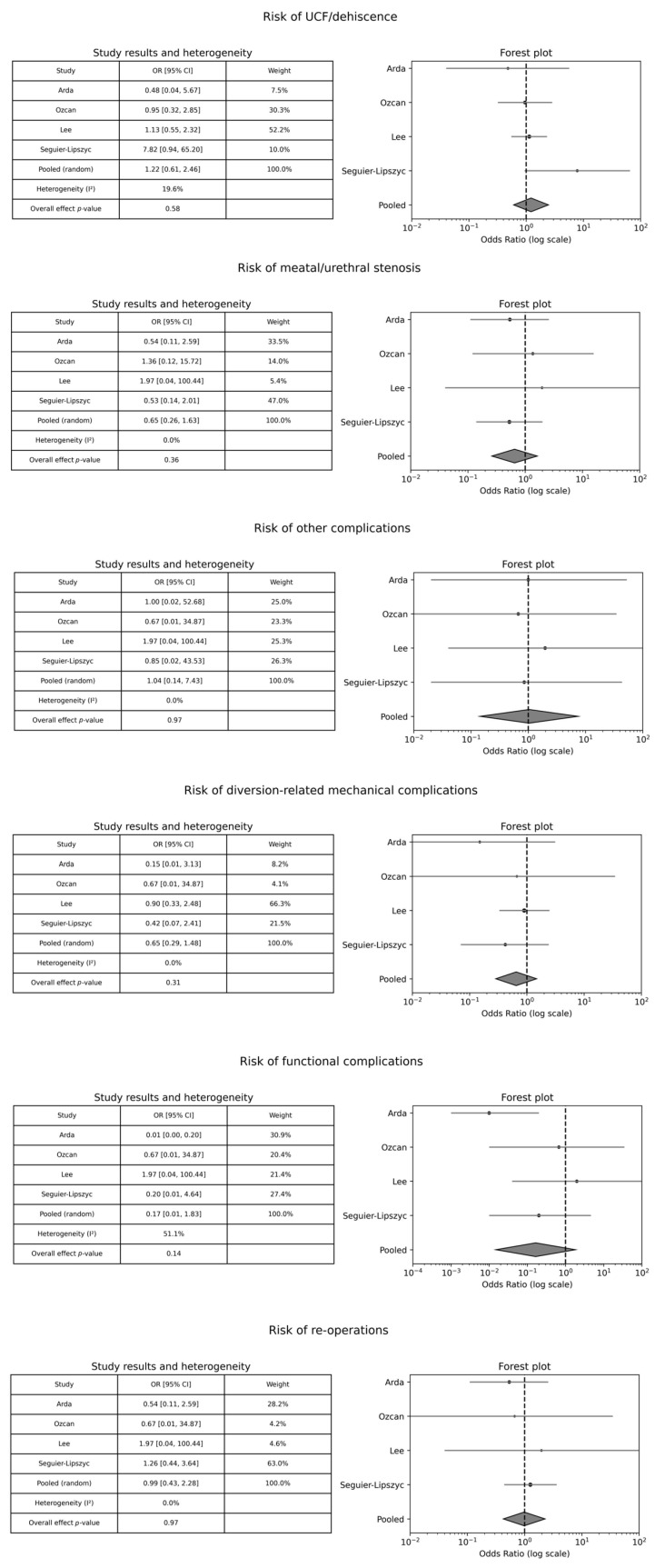
Forest plot comparison between bladder catheter versus urethral stent on surgical outcomes. OR: Odds Ratio; CI: Confidence Interval.

Four papers [10,24,28,38] compared the effect of bladder catheter versus urethral stent on surgical outcomes. The findings did not demonstrate a consistent advantage of one type of diversion over the other. The studies did not provide evidence for a difference in the risk of UCF/dehiscence, meatal/urethral stenosis, other complications, diversion-related mechanical complications, functional complications, and re-operations between the compared groups (Figure 2).

#### 3.4.2. Stented vs. Unstented Procedures

**Figure 3:**
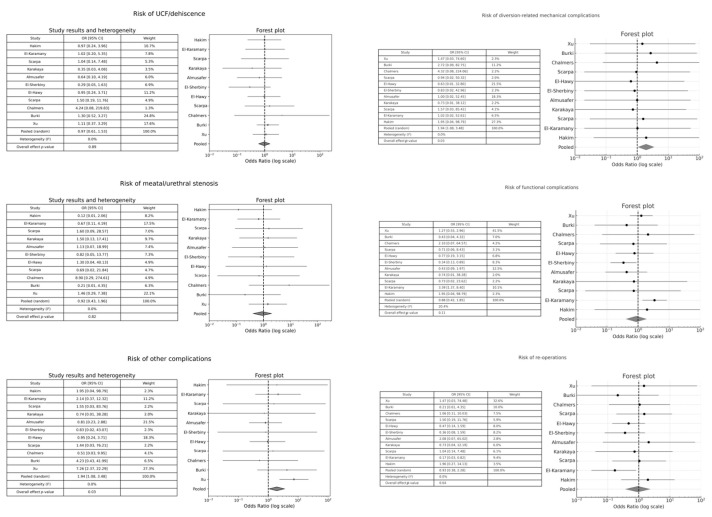
Forest plot comparison between stented versus unstented procedures on surgical outcomes.

Eleven studies [9,11,20,21,25,26,27,31,33,34,35] compared the effect of stented versus unstented procedures on surgical outcomes. In most analyses, the confidence intervals encompassed the null value (OR = 1), suggesting no statistically significant difference between the two techniques. Collectively, there was no evidence of a difference in the risk of UCF/dehiscence, meatal/urethral stenosis, diversion-related mechanical complications, functional complications, and re-operations between the comparison groups (Figure 3). Conversely, when considered together, the studies showed a statistically significant increase in the risk of other complications—WI, UTI, fever, penile swelling, and foreskin dehiscence—for stented procedures compared to unstented ones [pooled OR 1.94; 95% CI: 1.08–3.48; *p* = 0.03] (Figure 3).

These results suggested that routine stenting did not provide a clear benefit in reducing postoperative complications, and its use should instead be tailored to intraoperative findings and surgeon preference.

#### 3.4.3. Catheterization Length ≤ 5 Days vs. Catheterization Length > 5 Days

**Figure 4:**
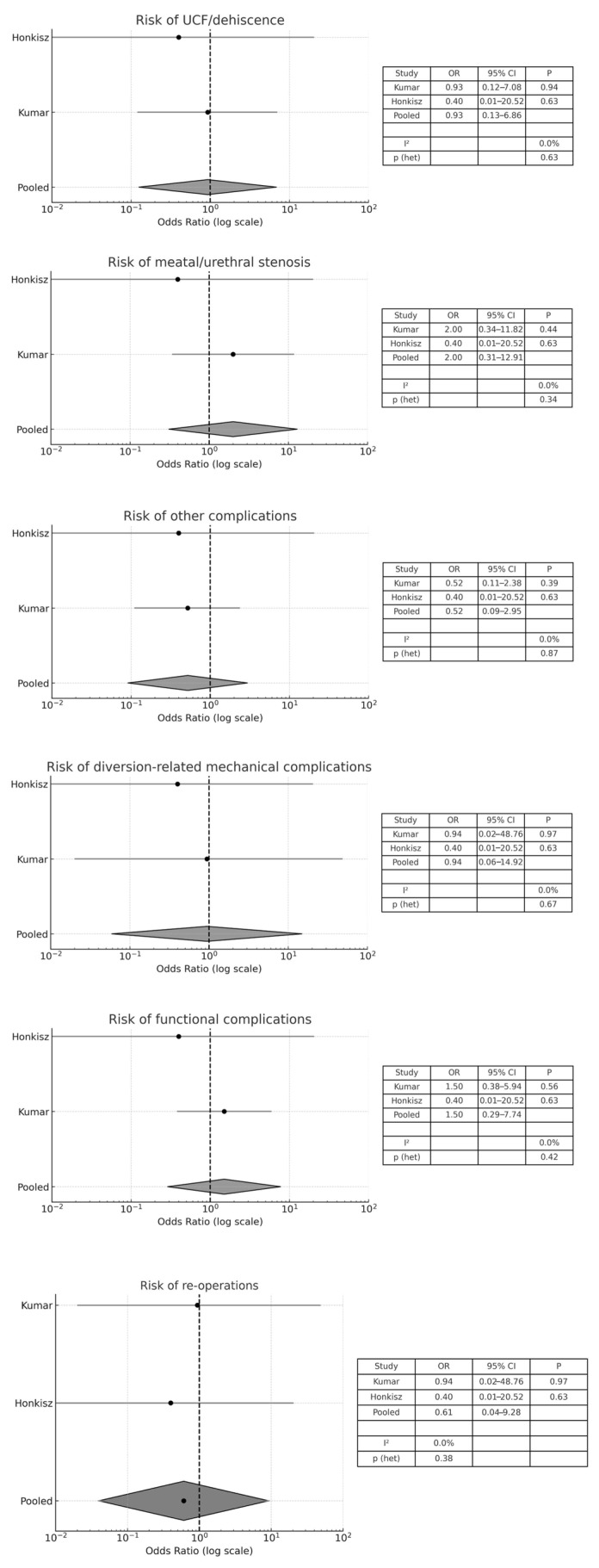
Forest plot comparison between catheter duration (≤5 days versus >5 days) on surgical outcomes.

Two studies [32,36] compared the effect of catheter duration (≤5 days vs. >5 days) on postoperative outcomes. Across all outcome categories, the pooled ORs with their respective 95% CIs suggested no statistically significant differences between shorter and longer catheterization. For example, the pooled OR for risk of re-operations was 0.61 (95% CI: 0.04–9.28; *p* = 0.38), for meatal/urethral stenosis it was 2.00 (95% CI: 0.31–12.91; *p* = 0.34), and for diversion-related mechanical complications it was 0.94 (95%CI: 0.06–14.92; *p* = 0.67). All meta-analyses showed I^2^ = 0.0%, indicating no observed heterogeneity between the included studies (Figure 4). These findings suggested that extending catheterization beyond five days did not provide a clear advantage in preventing postoperative complications and may be unnecessary. However, the wide confidence intervals highlighted the limited statistical power and variability of the included studies, as well as the need for future high-quality prospective trials to confirm these results.

### 3.5. Publication Bias

Assessment of publication bias is reported in Supplementary Figures S1–S3.

For the comparison between stented and unstented repairs, funnel plots for UCF/dehiscence, re-operation, and other complications did not reveal marked asymmetry (Supplementary Figure S1a–c). Egger’s regression test was non-significant for UCF/dehiscence (*p* = 0.22), re-operations (*p* = 0.67), and other complications (*p* = 0.17).

For the catheter vs. stent comparison, four comparative studies reported UCF/dehiscence, with Egger’s test showing no evidence of asymmetry (*p* = 0.36) (Supplementary Figure S2a), while re-operations were assessed in only two studies, precluding formal testing (Supplementary Figure S2b).

For the analysis of catheter duration (≤5 vs. >5 days), only one comparative study for UCF and two for other complications were available, preventing a meaningful Egger test. Funnel plots are nonetheless provided for transparency (Supplementary Figure S3a,b).

## 4. Discussion

Despite decades of experience and technical refinement, the optimal urinary diversion strategy following hypospadias repair remains a matter of ongoing debate, with considerable variability in clinical practice [5,6,7]. This systematic review and meta-analysis aimed to answer the following questions: (1) Which type of urinary diversion is the most effective? (2) Which material should be used? (3) What is the optimal duration of diversion? (4) Is urinary diversion necessary after hypospadias repair?

The study findings contributed to clarifying some such uncertain aspects, offering evidence-based guidance on the influence of urinary diversion methods on postoperative outcomes after hypospadias surgery.

Bladder catheters were associated with a higher risk of some non-specific postoperative complications, such as WI, UTI, penile swelling, and foreskin dehiscence, and re-operation. In contrast, urethral stents appeared to show a trend toward a safer profile, although the pooled analysis did not confirm significant differences for the major outcomes such as fistula or stenosis. However, urethral stents were associated with a higher incidence of mechanical issues, including obstruction and dislodgement. This finding suggested that while urethral stents may provide a more physiological urinary flow and minimize bladder irritation, their placement and maintenance required greater technical precision and postoperative vigilance to avoid mechanical failure. On the other side, the greater burden of systemic complications and re-operations observed in the bladder catheter group highlighted the importance of minimizing bladder manipulation whenever feasible, especially in less severe forms of hypospadias.

Regarding material composition, a low number of studies reported the type of material, including silicone, latex, polyvinyl chloride, C-flex, and silastic [11,17,20,22,23,24,27,28,35,37,38,39]. However, it is unclear whether there is a significant correlation between the material used and surgical outcomes.

Catheterization length emerged as another key factor influencing postoperative outcomes. Shorter catheterization (≤5 days) was associated with significantly higher rates of functional complications, such as bladder spasms, urinary retention, and extravasation, as well as an overall higher rate of other complications and re-operations. These findings may support the rationale for maintaining urinary diversion for more than five days in selected cases. However, longer catheterization (>5 days) was also linked to a significantly increased risk of UCF and glans dehiscence. This effect may be attributed to delayed inflammatory response or microtrauma at the repair site due to prolonged foreign body presence. Notably, when only studies directly comparing short vs. long catheterization were pooled [32,36], no significant differences emerged. This highlighted the divergence between descriptive data and pooled analyses, which must be interpreted with caution, given the limited number and heterogeneity of available studies. Moreover, our review confirmed the delicate balance between allowing sufficient time for neourethral stabilization and avoiding prolonged catheter-related irritation and trauma.

Regarding the need for urinary diversion following distal hypospadias repair, there is no consensus. For distal hypospadias, meatal localization and the patient’s toilet status were reported to be non-essential factors in the use and length of catheterization [14]. Some authors favored the use of a stent in distal hypospadias repair in toilet-trained children to avoid the risk of urinary retention and extravasation and reduce the overall patient discomfort and the need for re-operations [11]. Conversely, other authors supported a non-stented technique for distal hypospadias repair to simplify the postoperative care and obviate the need for antibiotics and anticholinergics [13,15,18,20,21]. Absence of urethral stent for distal hypospadias repair did not seem to be associated with increased risk of postoperative complications [13].

The comparison between stented and unstented procedures provided interesting findings. Stented procedures were associated with a statistically higher rate of other complications, particularly infections and swelling; however, pooled analyses did not show significant differences in major outcomes such as fistula, stenosis, or re-operations. This result aligned with previous reports questioning the necessity of routine stenting, especially in distal hypospadias repair [11]. The protective role of stents in maintaining postoperative urethral patency must be weighed against the added risk of complications and the potential discomfort for the patient. The review data suggest that routine stenting may not provide a clear benefit in reducing postoperative complications. A selective approach, tailored according to intraoperative findings and patient-specific factors, appears reasonable, although further studies are needed before definitive recommendations can be made. These conclusions are in line with the most recent EAU/ESPU Paediatric Urology Guidelines [8], which recognize transurethral stents, suprapubic drainage, or omission of drainage as acceptable options, particularly for distal repairs. Importantly, the guidelines emphasize that the decision should be individualized, as no single approach has been proven superior, and the duration of stenting remains without consensus [40].

Regarding other specific aspects of urinary diversion in hypospadias surgery, some experimental studies were focused on the optimization of use of silicone urinary catheters for hypospadias repair. Hardwicke et al. [41] advised a maximum instillation of 2 mL of water into a 5 mL pediatric catheter balloon to avoid cuff formation. This technique allows reductions in the risk of trauma and complications associated with catheter removal. In another experimental study [42], an all-silicone catheter inflated with 2 mL and removed from the urethra within 24–72 h was proposed as the ideal use of catheters in hypospadias surgery.

This systematic review is not without limitations. First, the included studies were heterogeneous in design, with many retrospective and non-randomized cohorts, potentially introducing risk of selection and reporting bias. The lack of randomized controlled trials further limited the strength of the evidence and increased the risk of confounding. Second, considerable variation existed in the definition of outcomes, catheter materials used, and surgical techniques applied. Third, despite applying strict inclusion criteria and performing subgroup analyses, residual confounding factors cannot be excluded. Despite these limitations, the study followed a robust methodology in accordance with PRISMA guidelines and included a comprehensive dataset, which strengthened the reliability of the findings, although results should still be interpreted with caution. Moreover, while formal sensitivity analyses were not feasible due to the limited number of comparative studies and variability of reported outcomes, we carefully performed subgroup analyses (by diversion type and duration) to mitigate the effect of heterogeneity and enhance interpretability.

From a clinical perspective, the findings of this review suggest that the choice of urinary diversion following hypospadias repair should be individualized rather than routine. Surgeons should carefully balance the potential advantages of stents in reducing systemic complications against their risk of mechanical issues, while also considering patient age, severity of hypospadias, and intraoperative findings. Similarly, the duration of catheterization should be tailored to achieve adequate tissue healing while minimizing device-related morbidity. In the absence of high-quality randomized evidence, our results may support a more selective and patient-based approach, encouraging surgeons to move away from ‘one-size-fits-all’ strategies and instead adopt diversion practices adapted to each clinical situation.

## 5. Conclusions

This systematic review and meta-analysis highlighted that no single urinary diversion strategy after hypospadias repair was universally superior. While bladder catheters and urethral stents each carried specific risks, pooled data showed no clear advantage in preventing major complications such as fistula or stenosis. Duration of diversion also represented a delicate balance between sufficient neourethral stabilization and device-related morbidity. Shorter catheterization was linked to increased risk of both functional and general postoperative complications, as well as re-operation. However, prolonged catheterization increased the risk of urethrocutaneous fistula. These findings support the need for a selective, individualized approach to perioperative management, tailored to patient characteristics, hypospadias severity, and intraoperative considerations, rather than a one-size-fits-all strategy. Given the heterogeneity of available studies and lack of randomized data, further high-quality prospective studies are warranted to define the optimal urinary diversion strategy to improve surgical outcomes in pediatric hypospadias repair.

## Data Availability

The data published in this study are available upon request from the corresponding author.

## Supplementary Materials

The following supporting information can be downloaded at: https://www.mdpi.com/article/10.3390/medicina61091659/s1, Table S1: Newcastle-Ottawa Scale (NOS) assessment of included studies; Table S2: Study and patient characteristics; Table S3: Surgical outcomes between different types of urinary diversion (bladder catheter vs. urethral stent); Table S4: Surgical outcomes between stented vs. unstented procedures; Table S5: Surgical outcomes between different lengths of catheterization (≤5 days vs. >5 days); Figure S1: Funnel plots for stented vs. unstented repairs; Figure S2: Funnel plots for bladder catheter vs. urethral stent; Figure S3: Funnel plots for catheter duration (≤5 vs. >5 days).

## Abbreviations

The following abbreviations are used in this manuscript:

PRISMA	Preferred Reporting Items for Systematic Reviews and Meta-analyses
RCT	Randomized controlled trial
UCF	Urethrocutaneous fistula
AUR	Acute urinary retention
OR	Odds ratio
CI	Confidence interval
WI	Wound infection
UTI	Urinary tract infection
