# Supplementary material for: The Impact of Postoperative Urinary Diversion on Surgical Outcomes of Hypospadias Repair: A Systematic Review and Meta-Analysis of Pediatric Literature
Source: Medicina (Kaunas). 2025 Sep 12;61(9):1659. doi: 10.3390/medicina61091659 (PMC12471891; doi:10.3390/medicina61091659)
Supplement: Supplementary file 1 [file medicina-61-01659-s001.zip › Supplementary Table S1.pdf]

Supplementary Table S1. Newcastle-Ottawa Scale (NOS) assessment of included studies

Author/Year	Study Design	Selection (0–4)	Comparability (0–2)	Outcome (0–3)	Total Score (0–9)	Quality Rating*
Hakim[9] 1996	Retrospective observational	3	1	2	6	Moderate
Arda[10] 2001	Prospective RCT	4	2	3	9	High
El-Sherbiny[11] 2003	Retrospective cohort	3	1	2	6	Moderate
Lorenz[12] 2004	Prospective observational	3	1	2	6	Moderate
Leclair[13] 2004	Retrospective observational	3	1	2	6	Moderate
Aslan[14] 2007	Retrospective observational	2	1	2	5	Moderate
Almodhen[15] 2008	Prospective observational	3	1	2	6	Moderate
Ritch[16] 2010	Retrospective observational	2	1	2	5	Moderate
Chang[17] 2011	Retrospective observational	2	1	2	5	Moderate
Turial[18] 2011	Prospective observational	3	1	2	6	Moderate
Radwan[19] 2012	Prospective RCT	4	2	3	9	High
Xu[20] 2013	Retrospective observational	2	1	2	5	Moderate
Chalmers[21] 2014	Prospective comparative	3	2	2	7	High
Polat[22] 2015	Retrospective observational	2	1	2	5	Moderate
Daher[23] 2015	Retrospective observational	2	1	2	5	Moderate
Ozcan[24] 2017	Prospective non-RCT	3	1	2	6	Moderate
Karakaya[25] 2017	Retrospective comparative	2	1	2	5	Moderate
El-Karamany[26] 2017	Prospective RCT	4	2	3	9	High
Scarpa[27] 2017	Retrospective observational	2	1	2	5	Moderate
Lee[28] 2018	Prospective non-RCT	3	1	2	6	Moderate
Sarac[29] 2018	Retrospective observational	2	1	2	5	Moderate
Assadi[30] 2020	Retrospective observational	2	1	2	5	Moderate
Almusafer[31] 2020	Prospective cross-sectional	3	1	2	6	Moderate
Honkisz[32] 2020	Retrospective comparative	2	1	2	5	Moderate

Author/Year	Study Design	Selection (0–4)	Comparability (0–2)	Outcome (0–3)	Total Score (0–9)	Quality Rating*
Scarpa[33] 2021	Retrospective cohort	2	1	2	5	Moderate
El-Hawy[34] 2021	Retrospective observational	2	1	2	5	Moderate
Burki[35] 2022	Retrospective observational	2	1	2	5	Moderate
Kumar[36] 2022	Prospective cohort	3	1	2	6	Moderate
Zhou S.[37] 2024	Retrospective cohort	3	1	2	6	Moderate
Seguier-Lipszyc[38] 2024	Retrospective observational	2	1	2	5	Moderate
Zhou G.[39] 2024	Prospective observational	3	1	2	6	Moderate

RCT=randomized clinical trial

*Quality rating: High (7–9), Moderate (5–6), Low (≤4).
