# Supplementary material for: The Impact of Postoperative Urinary Diversion on Surgical Outcomes of Hypospadias Repair: A Systematic Review and Meta-Analysis of Pediatric Literature
Source: Medicina (Kaunas). 2025 Sep 12;61(9):1659. doi: 10.3390/medicina61091659 (PMC12471891; doi:10.3390/medicina61091659)
Supplement: Supplementary file 1 [file medicina-61-01659-s001.zip › Supplementary Table S2.pdf]

Supplementary Table S2. Study and patient characteristics

Author /Year	Study Design	Series, n=	Patient age, median	Hypospadias Degree (n=)	Surgical Technique (n=)	Urinary Diversion (n=)
Hakim[9] 1996	Retrospective observational	336	19.9	Distal (336)	Modified Mathieu (336)	Group 1-2: unstented (222)<br>Group 3-4: urethral stent (114)
Arda[10] 2001	Prospective randomized clinical trial	44	48	Distal (26)<br>Mid-penile (18)	Mathieu (26)<br>Preputial island flap (18)	Group 1: feeding tube as stent (22)<br>Group 2: feeding tube as catheter (22)
El-Sherbiny[11] 2003	Retrospective cohort	64	n/a	Distal (64)	TIPU (64)	Group 1: silicon catheter (35)<br>Group 2: unstented (29)
Lorenz[12] 2004	Prospective observational	27	37.2	Distal (22)<br>Midshaft (5)	TIPU (27)	Urethral catheter (27)
Leclair[13] 2004	Retrospective observational	162	11.3	Glanular (7)<br>Coronal/ subcoronal (149)<br>Mid-shaft (6)	TIPU (162)	Unstented (162)
Aslan[14] 2007	Retrospective observational	128	40.4	Distal (128)	TIPU (128)	Feeding tube (128)
Almodhen[15] 2008	Prospective observational	32	18	Distal + mid-shaft (26)<br>Proximal (6)	TIPU (32)	None
Ritch[16] 2010	Retrospective observational	49	14.8	Distal (39)<br>Midshaft (10)	TIPU (49)	Urethral catheter (49)
Chang[17] 2011	Retrospective observational	86	29.5	Distal (58)<br>Midshaft (15)<br>Proximal (13)	TIPU (58)<br>Mathieu (15)<br>Duckett procedure (13)	Group 1: silicone stent (23)<br>Group 2: double pigtail stent (63)

Turial[18] 2011	Prospective observational	41	36	Coronal (25)<br>Mid-shaft (16)	TIPU (41)	None
Radwan[19] 2012	Prospective randomized clinical trial	192	52	Distal (192)	TIPU (192)	Group 1: urethral stent (63)<br>Group 2: suprapubic catheter (63)<br>Group 3: suprapubic catheter + small catheter in the anterior urethra (66)
Xu[20] 2013	Retrospective observational	254	52.2	Distal (167)<br>Mid-shaft (87)	TIPU (254)	Group 1: silicone urethral catheter (103)<br>Group 2: unstented (151)
Chalmers[21] 2014	Prospective comparative	110	7	Distal (110)	TIPU (103)<br>MAGPI (7)	Group 1: feeding tube (21)<br>Group 2: unstented (89)
Polat[22] 2015	Retrospective observational	35	36.2	Distal (22)<br>Midshaft (13)	TIPU (35)	Foley latex (35)
Daher[23] 2015	Retrospective observational	189	17	Distal (135)<br>Mid-shaft (23)<br>Proximal (31)	Modified Duplay (174)<br>Duckett (15)	Polyvinyl chloride entering feeding tube (189)
Ozcan[24] 2017	Prospective non-randomized clinical trial	77	40.6	Distal (77)	TIPU (77)	Group 1: Zaontz urethral stent (31)<br>Group 2: feeding tube (46)
Karakaya[25] 2017	Retrospective comparative	66	28.5	Distal (66)	TIPU (66)	Group 1: urethral stent (38)<br>Group 2: unstented (28)
El-Karamany[26] 2017	Prospective randomized clinical trial	93	38	Distal (93)	TIPU (93)	Group 1: feeding tube (46)<br>Group 2: unstented (47)

Scarpa[27] 2017	Retrospective observational	44	25.5	Distal (44)	TIPU (44)	Group 1: silicon bladder catheter for 6 days (18)<br>Group 2: silicon bladder catheter for 1 day (26)
Lee[28] 2018	Prospective non-randomized clinical trial	150	12	Distal (98)<br>Midshaft + proximal (52)	TIPU (124) MAGPI, GAP or other (11)<br>Second-stage urethroplasty (15)	Group 1: silastic tube (76)<br>Group 2: Koyle stent (74)
Sarac[29] 2018	Retrospective observational	123	63.2	Coronal (50)<br>Subcoronal (73)	TIPU (123)	Group 1: Foley catheter<br>(32)<br>Group 2: feeding tube (91)
Assadi[30] 2020	Retrospective observational	195	7.4	Distal (94)<br>Midshaft (48)	TIPU (142)	Unstented (142)
Almusafer[31] 2020	Prospective cross-sectional	50	73.2	Distal (50)	TIPU (50)	Group 1: urethral stent (25)<br>Group 2: unstented (25)
Honkisz[32] 2020	Retrospective comparative	95	23.3	Distal (95)	Advanced MAGPI (95)	Group 1: bladder catheter for 0–1 day (33)<br>Group 2: bladder catheter for >5 days (27)<br>Group 3: bladder catheter for 0-1 day (35)
Scarpa[33] 2021	Retrospective cohort	28	24	Coronal/ subcoronal (28)	TIPU (28)	Group 1: Foley catheter (11)<br>Group 2: unstented (17)
El-Hawy[34] 2021	Retrospective observational	72	18.9	Distal (72)	TIPU (72)	Group 1: bladder catheter (44)<br>Group 2: unstented (28)

Burki[35] 2022	Retrospective observational	120	24.5	Coronal/ subcoronal (101)<br>Mid-shaft (19)	TIPU/ Thiersch-Duplay (120)	Group 1: unstented (63)<br>Group 2: Zaontz or feeding tube (57)
Kumar[36] 2022	Prospective cohort	62	60	Distal (41)<br>Mid-proximal (21)	TIPU (62)	Group 1: urethral catheter (32) for <5 days<br>Group 2: urethral catheter (32) for >5 days
Zhou S[37] 2024	Retrospective cohort	576	24	Penile (206)<br>Penoscrotal + Scrotal (355)<br>Perineal (15)	One-stage Duckett procedure (576)	Group 1: NTAS over silicone catheter (398)<br>Group 2: silicone stent over silicone catheter (178)
Seguier-Lipszyc[38] 2024	Retrospective observational	96	59.4	Distal (96)	TIPU (27)<br>MAGPI (18)<br>Fistula repair (25)<br>Meatoplasty (22)<br>Urethral advancement (4)	Zaontz stent (96)
Zhou G[39] 2024	Prospective observational	665	35.6	Proximal with severe curvature (665)	Duckett procedure (145)<br>Staged urethroplasty (520)	Urethral stent ± silicone single-lumen bladder catheter (665)
