# Supplementary material for: The Impact of Postoperative Urinary Diversion on Surgical Outcomes of Hypospadias Repair: A Systematic Review and Meta-Analysis of Pediatric Literature
Source: Medicina (Kaunas). 2025 Sep 12;61(9):1659. doi: 10.3390/medicina61091659 (PMC12471891; doi:10.3390/medicina61091659)
Supplement: Supplementary file 1 [file medicina-61-01659-s001.zip › Supplementary Table S3.pdf]

Supplementary Table S3. Surgical outcomes between different types of urinary diversion (bladder catheter vs urethral stent)

**BLADDER CATHETER**								
**Author/Year**	**Patient number** n= (overall)	**Urinary diversion** Type (n=)	**UCF/ dehiscence** n (%)	**Meatal/ urethral stenosis** n (%)	**Other** n (%)	**Diversion-related mechanical complications** n (%)	**Functional complications** n (%)	**Re-operations** n (%)
Arda[10] 2001	22 (44)	Feeding tube (22)	1 (4.5)	3 (13.6)	0	0	0	3 (13.6) dilatation
El-Sherbiny[11] 2003	35 (64)	Silicon catheter	2 (5.7)	1 (2.8)	0	0	4 (9.1) bladder spasm<br>1 (2.3) AUR	3 (8.6) redo-surgery
Lorenz[12] 2004	27	Urethral catheter (27)	1 (3.7)	2 (7.4)	0	0	0	2 (7.4) urethrotomy ± fistula closure<br>1 (3.7) redo-urethroplasty
Aslan[14] 2007	128	Feeding tube (128)	10 (7.8)	3 (2.3)	2 (1.6) buried penis	0	0	10 (7.8) fistula closure ± redo-urethroplasty<br>3 (2.3) meatotomy
Ritch[16] 2010	49	Bladder catheter (49)	5 (10.2)	0	0	0	0	1 (2) persistent chordee
Xu[20] 2013	103	Silicone urethral catheter	6 (5.8)	3 (2.9)	7 (6.7) WI<br>10 (9.7) UTI	0	11 (10.7) bladder spasms	n/a
Chalmers[21] 2014	21 (110)	Feeding tube	0	1 (4.76)	0	0	0	1 (4.76) redo-surgery

Polat[22] 2015	35	Foley latex (35)	1 (2.8%)	0	2 (5.7%) UTI	0	0	n/a
Daher[23] 2015	189	Polyvinyl chloride entering feeding tube (189)	22 (11.6)	6 (3.2)	0	0	0	22 (11.6) fistula closure<br>6 (3.2) meatotomy
Ozcan[24] 2017	46 (77)	Feeding tube (46)	10 (21.7)	2 (4.3)	0	0	0	n/a
El-Karamany [26] 2017	46 (93)	Feeding tube (46)	3 (6.5)	2 (4.3)	3 (6.5) WI<br>1 (2.2) hematoma	0	22 (48) bladder spasm	5 (10.8)
Scarpa[27] 2017	18 (44)	Silicon bladder catheter	2 (11.1)	0	0	0	1 (5.5) AUR	2 (11.1) redo-surgery
Lee[28] 2018	76 (150)	Silastic tube (76)	14 (21)	n/a	0	6 (8) blockage kinking	0	n/a
Sarac[29] 2018	123	Foley (32)<br>Feeding tube (91)	14 (11.4)	0	0	0	0	4 (3.25)
Honkisz[32] 2020	95	Bladder catheter	11 (11.6)	n/a	0	n/a	0	n/a
Scarpa[33] 2021	11 (28)	Foley catheter	2 (18.2)	1 (9.1)	0	0	0	2 (18.2) redo-urethroplasty
El-Hawy[34] 2021	44 (72)	Bladder catheter	6 (13.6)	1 (2.3)	6 (13.6) WI	0	4 (9.1) bladder spasm<br>1 (2.3) AUR	6 (13.6) redo-surgery
Kumar[36] 2022	62	Urethral catheter (62)	4 (6.4)	6 (9.7)	4 (6.4) UTI<br>4 (6.4) WI	0	4 (6.4) bladder spasm<br>6 (9.7) urinary retention/ extravasation	n/a
Seguier-Lipszyc[38] 2024	52 (96)	Foley catheter ± urethral stent in bladder	8 (15.4)	4 (7.7)	0	2 (3.8) catheter obstruction	0	10 (19.2)
**Total**	1182		122	35	39	8	54	81

			(10.3%)	(2.9%)	(3.3%)	(0.7%)	(4.6%)	(6.8%)

URETHRAL STENT

**Author/Year**	**Patient number** n= (overall)	**Urinary diversion** Type (n=)	**UCF/ dehiscence** n (%)	**Meatal/ urethral stenosis** n (%)	**Other** n (%)	**Diversion-related mechanical complications** n (%)	**Functional complications** n (%)	**Re-operations** n (%)
Hakim[9] 1996	114 (336)	Urethral stent	3 (2.63)	0	0	0	0	2 (1.75)
Arda[10] 2001	22 (44)	Feeding tube used as stent (22)	2 (13.6)	5 (22.7%	0	3 (13.6) stent dislodgement	22 (100) straining at 1st voiding<br>19 (86.4) pain at 1st voiding<br>2 (9.1) AUR	5 (22.7) dilatation
Chang[17] 2011	86	Silicone stent (23)<br>Double pigtail stent (63)	15 (17.4)	0	0	6 (7) stent dislodgement + wound disruption	0	n/a
Radwan[19] 2012	63 (192)	Urethral stent (63)	11 (17.4)	3 (4.7)	0	0	21 (33) bladder spasm	n/a
Ozcan[24] 2017	31 (77)	Zaontz stent	7 (22.5)	1 (3.2)	0	0	0	n/a
Karakaya[25] 2017	38 (66)	Urethral stent	1 (2.6)	2 (5.2)	0	0	0	1 (2.6) redo-surgery
Lee[28] 2018	74 (150)	Koyle stent (74)	25 (16.7)	0	0	13 (8.7) blockage/ kinking	0	n/a
Almusafer [31] 2020	25 (50)	Urethral stent	2 (8)	1 (4)	2 (8) UTI<br>1 (4) WI<br>3 (12) fever	0	3 (12) dysuria	1 (4) fistula closure
Burki[35] 2022	47 (120)	Zaontz or feeding tube	11 (23.4)	0	3 (5.1) penile swelling	1 (1.7) catheter blockage	1 (1.7) bladder spasm	n/a

Zhou S.[37] 2024	576	NTAS over silicone catheter (398) Silicone stent over silicone catheter (178)	65 (11.3)	7 (1.2)	0	0	0	n/a
Seguier-Lipszyc[38] 2024	44 (96)	Zaontz stent	1 (2.3)	6 (13.6)	0	2 (4.6) stent dislodgement	2 (4.6) AUR	7 (15.9)
**Total**	1120		143 (12.8%)	25 (2.2%)	9 (0.8%)	25 (2.2%)	70 (6.2%)	16 (1.4%)
**Odds Ratio (OR)**			0.80	1.34	4.21	0.30	0.72	5.08
**Lower 95% CI**			0.61	0.79	2.03	0.16	0.50	2.95
**Upper 95% CI**			1.02	2.25	8.74	0.66	1.03	8.73
**Chi-square**			3.38	1.20	17.55	9.84	3.19	41.92
**P value**			0.07	0.27	**<0.05**	**0.001**	0.07	**<0.05**

UCF=urethrocutaneous fistula; WI=wound infection; UTI=urinary tract infection; AUR=acute urinary retention; n/a=not available
