# Supplementary material for: The Impact of Postoperative Urinary Diversion on Surgical Outcomes of Hypospadias Repair: A Systematic Review and Meta-Analysis of Pediatric Literature
Source: Medicina (Kaunas). 2025 Sep 12;61(9):1659. doi: 10.3390/medicina61091659 (PMC12471891; doi:10.3390/medicina61091659)
Supplement: Supplementary file 1 [file medicina-61-01659-s001.zip › Supplementary Table S4.pdf]

Supplementary Table S4. Surgical outcomes between stented vs unstented procedures

STENTED PROCEDURES

**Author/Year**	**Patient number** n= (overall)	**Urinary diversion** Type (n=)	**UCF/ dehiscence** n (%)	**Meatal/ urethral stenosis** n (%)	**Other** n (%)	**Diversion-related mechanical complications** n (%)	**Functional complications** n (%)	**Re-operations** n (%)
Hakim[9] 1996	114 (336)	n/a	3 (2.63)	0	0	0	0	2 (1.75)
El-Sherbiny[11] 2003	35 (64)	Silicon catheter	2 (5.7)	1 (2.8)	0	0	3 (8.6) bladder spasm<br>5 (14.3) dysuria	3 (8.6) redo-surgery
Xu[20] 2013	103 (254)	Urethral catheter	6 (5.8)	3 (2.9)	7 (6.7) WI<br>10 (9.7) UTI	0	11 (10.7) bladder spasm	n/a
Chalmers[21] 2014	21 (110)	Feeding tube	0	1 (4.76)	0	0	0	1 (4.76) redo-surgery
Karakaya[25] 2017	38 (66)	n/a	1 (2.6)	2 (5.2)	0	0	0	1 (2.6) redo-surgery
El-Karamany[26] 2017	46 (93)	Feeding tube (46)	3 (6.5)	2 (4.3)	3 (6.5) WI<br>1 (2.2) hematoma	0	22 (48) bladder spasm	5 (10.8)
Scarpa[27] 2017	18 (44)	Silicon bladder catheter	2 (11.1)	0	0	0	1 (5.5) AUR	2 (11.1) redo-surgery
Almusafer[31] 2020	25 (50)	n/a	2 (8)	1 (4)	2 (8) UTI<br>1 (4) WI<br>3 (12) fever	0	3 (12) dysuria	1 (4) fistula closure
Scarpa[33] 2021	11 (28)	Foley catheter	2 (18.2)	1 (9.1)	0	0	0	2 (18.2) redo-surgery
El-Hawy[34] 2021	44 (72)	Bladder catheter	6 (13.6)	1 (2.3)	6 (13.6) WI	0	4 (9.1) bladder spasm	6 (13.6) redo-surgery

							1 (2.3) AUR	
Burki[35] 2022	47 (120)	Zaontz or feeding tube	11 (23.4)	0	3 (5.1) penile swelling	1 (1.7) catheter blockage	1 (1.7) bladder spasm	n/a
Seguier-Lipszyc[38] 2024	96	Zaontz stent	9 (9.4)	10 (10.4)	0	2 (3.8) catheter obstruction	2 (3.8) stent dislodgement<br>2 (3.8) AUR	17 (17.7)
**Total**	598		47 (7.8%)	22 (3.7%)	36 (6.0%)	3 (0.5%)	55 (9.2%)	40 (6.7%)
**UNSTENTED PROCEDURES**								
**Author/Year**	**Patient number** n= (overall)	**Urinary diversion** Type (n=)	**UCF/ dehiscence** n (%)	**Meatal/ urethral stenosis** n (%)	**Other** n (%)	**Diversion-related mechanical complications** n (%)	**Functional complications** n (%)	**Re-operations** n (%)
Hakim[9] 1996	222 (336)	None	6 (2.7)	8 (3.6)	0	n/a	0	1 (0.45) redo-urethroplasty<br>1 (0.45) meatotomy
El-Sherbiny[11] 2003	29 (64)	None	5 (17.2)	1 (3.4)	0	n/a	13 (44.8) dysuria<br>7 (24.1) AUR<br>5 (17.2) extravasation	6 (20.7) redo-surgery
Leclair[13] 2004	162	None	9 (5.6)	4 (2.5)	6 (4.4) foreskin dehiscence<br>13 (9.5) secondary phimosis	n/a	4 (2.5) AUR	8 (4.9) redo-surgery<br>2 (1.2) meatal dilatation<br>2 (1.2) meatoplasty<br>2 (1.2) circumcision<br>2 (1.2) redo foreskin

								reconstruction<br>2 (1.2) secondary preputioplasty
Almodhen[15] 2008	32	None	0	1 (3.1)	1 (3.1) pyelonephritis	n/a	1 (3.1) extravasation	1 (3.1) office meatotomy
Turial[18] 2011	41	None	2 (4.9)	1 (2.4)	2 (4.9) foreskin dehiscence	n/a	0	2 (4.9) fistula repair<br>1 (2.4) urethral dilatation
Xu[20] 2013	151 (254)	None	8 (5.3)	5 (3.3)	4 (2.6) WI<br>5 (3.3) UTI	n/a	6 (4.0) AUR<br>2 (1.3) extravasation	n/a
Chalmers[21] 2014	89 (110)	None	0	0	4 (4.49) redundant shaft skin	n/a	1 (1.12) AUR	4 (4.49) circumcision
Karakaya[25] 2017	28 (66)	None	2 (7.1)	1 (3.6)	0	n/a	0	1 (3.6) urethral dilation
El-Karamany[26] 2017	47 (93)	None	3 (6.4)	3 (6.4)	1 (2.1) hematoma<br>1 (2.1) WI	n/a	4 (8.5) bladder spasms<br>6 (13) AUR	10 (21.3)
Scarpa[27] 2017	26 (44)	None	2 (7.7)	1 (3.8)	0	n/a	2 (7.7) AUR	2 (7.7) redo-surgery
Assadi[30] 2018	142 (195)	None	12/142 (8.5%)	6/142 (4.2%)	0	n/a	6/195 (3) multiple/split stream<br>2/195 (1) dysuria<br>2/195 (1) AUR<br>1/195 (0.5) hematuria	n/a
Almusafer[31] 2020	25 (50)	None	3 (12)	1 (4)	3 (12) UTI<br>4 (16) fever	n/a	2 (8) AUR<br>4 (16) dysuria	n/a

Scarpa[33] 2021	17 (28)	None	3 (17.6)	1 (5.9)	0	n/a	1 (5.9) AUR	3 (17.6) redo-urethroplasty
El-Hawy[34] 2021	28 (72)	None	4 (14.3)	0	4 (14.3) WI	n/a	4 (14.3) AUR	4 (14.3) redo-surgery<br>3 (10.7) suprapubic diversion
Burki[35] 2022	63 (120)	None	12/51 (23.5)	3/51 (5.8)	1 (1.6) bleeding	n/a	1/63 (1.6) AUR<br>2/63 (3.2) dysuria	3/51 (5.8) meatoplasty
**Total**	1102		71<br>(6.4%)	36<br>(3.3%)	49<br>(4.4%)	n/a	76<br>(6.9%)	60<br>(5.4%)
**Odds ratio (OR)**			1.24	1.13	1.38	11.1	1.37	1.24
**Lower 95% CI**			0.84	0.66	0.88	0.56	0.95	0.82
**Upper 95% CI**			1.82	1.94	2.14	222.02	1.96	1.88
**Chi-square**			1.20	0.2	2.02	3.93	2.88	1.08
**P value**			0.27	0.65	0.16	**0.05**	0.09	0.3

UCF=urethrocutaneous fistula; WI=wound infection; UTI=urinary tract infection; AUR=acute urinary retention; n/a=not available
