# Supplementary material for: The Impact of Postoperative Urinary Diversion on Surgical Outcomes of Hypospadias Repair: A Systematic Review and Meta-Analysis of Pediatric Literature
Source: Medicina (Kaunas). 2025 Sep 12;61(9):1659. doi: 10.3390/medicina61091659 (PMC12471891; doi:10.3390/medicina61091659)
Supplement: Supplementary file 1 [file medicina-61-01659-s001.zip › Supplementary Table S5.pdf]

Supplementary Table S5. Surgical outcomes between different lengths of catheterization (≤ 5 days vs > 5 days)

CATHETERIZATION LENGTH ⩽ 5 DAYS

**Author/Year**	**Patient number** n= (overall)	**Time of removal** average, days (range)	**UCF/ dehiscence** n (%)	**Meatal/ urethral stenosis** n (%)	**Other** n (%)	**Diversion-related mechanical complications** n (%)	**Functional complications** n (%)	**Re-operations** n (%)
Arda[10] 2001	44	1.5 (0-3)	3 (6.8)	8 (18.2)	0	3 (6.8) stent dislodgement	22 (50) straining at 1st voiding<br>19 (43.2) pain at 1st voiding<br>2 (4.5) AUR	8 (18.1) dilatation
Aslan[14] 2007	128	3.5 (0-7)	10 (7.8)	3 (2.3)	2 (1.6) buried penis	0	0	10 (7.8) fistula closure ± redo-urethroplasty<br>3 (2.3) meatotomy
Ritch[16] 2010	49	1	5 (10.2)	0	0	0	0	1 (2) persistent chordee
Polat[22] 2015	35	1.5 (1-2)	1 (2.8)	0	2 (5.7) UTI	0	0	n/a
Honkisz[32] 2020	68 (95)	0.5 (0-1)	n/a	n/a	9 (13.2) foreskin dehiscence	n/a	n/a	n/a
Kumar[36] 2022	32 (62)	≤5	2 (6.3)	4 (12.5)	1 (3.1) UTI<br>2 (6.3) WI	0	6 (18.7) AUR/ extravasation	n/a
**Total**	356	2.16	21 (5.9%)	15 (4.2%)	16 (4.5%)	3 (0.8%)	49 (13.8%)	22 (6.2%)

CATHETERIZATION LENGTH > 5 DAYS

**Author/Year**	**Patient number** n=	**Time of removal**	**UCF/ dehiscence**	**Meatal/ urethral stenosis**	**Other** n (%)	**Diversion-related**	**Functional complications**	**Re-operations** n (%)

	(overall)	average, days (range)	n (%)	n (%)		**mechanical complications** n (%)	n (%)	
Lorenz[12] 2004	27	13 (12-14)	1 (3.7)	2 (7.4)	0	0	0	2 (7.4) urethrotomy ± fistula closure<br>1 (3.7) redo-urethroplasty
Chang[17] 2011	86	8.5 (7-10)	15 (17.4)	0	0	6 (7) stent dislodgement + wound disruption	0	n/a
Radwan[19] 2012	63 (192)	6-7	11 (17.4)	3 (4.7)	0	0	21 (33) bladder spasm	n/a
Xu[20] 2013	103 (254)	7	6 (5.8)	3 (2.9)	7 (6.7) WI<br>10 (9.7) UTI	0	11 (10.7) bladder spasm	n/a
Daher[23] 2015	189	14 (7-21)	22 (11.6)	6 (6.7)	0	0	0	22 (11.6) fistula closure<br>6 (6.7) meatotomy
Ozcan[24] 2017	77	7	17 (22)	3 (3.8)	0	0	0	n/a
Lee[28] 2018	150	8.5 (7-10)	25 (16.7)	n/a	0	13 (8.7) blockage /kinking	0	n/a
Sarac[29] 2018	123	7	14 (11.4)	0	0	0	0	4 (3.25)
Honkisz[32] 2020	27 (95)	7<br>(5-9)	n/a	n/a	2 (7.4) foreskin dehiscence	0	n/a	n/a
Kumar[36] 2022	30 (62)	>5	2 (6.7)	2 (6.7)	3 (10) UTI<br>2 (6.7) WI	0	4 (13.3) bladder spasm	n/a
Zhou S.[37] 2024	576	28	65 (11.3)	7 (1.2)	0	0	n/a	n/a
Seguier-Lipszyc[38] 2024	96	7<br>(5.5-8)	9 (9.3)	10 (10.4)	0	2 (2) catheter obstruction<br>2 (2) stent dislodgement	2 (2) urinary retention	17 (17.7)

Zhou G.[39] 2024	665	27.7 (14-42)	138 (20.7)	42 (6.3)	15 (2.2) urethral diverticulum	0	0	11 (1.6) urethrotomy 7 (1) redo-urethroplasty
**Total**	2212	11.2	325 (14.7%)	78 (3.5%)	39 (1.8%)	23 (1.0%)	38 (1.7%)	70 (3.2%)
**Odds ratio (OR)**			0.36	1.2	2.62	0.81	9.13	2.02
**Lower 95% CI**			0.23	0.68	1.45	0.24	5.88	1.23
**Upper 95% CI**			0.57	2.12	4.74	2.71	14.18	3.3
**Chi-square**			20.34	0.41	10.91	0.12	135.95	8.07
**P value**			**<0.05**	0.52	**<0.05**	0.73	**<0.05**	**0.004**

UCF=urethrocutaneous fistula; WI=wound infection; UTI=urinary tract infection; AUR=acute urinary retention; n/a=not available
