# Supplementary figures and images for: The Impact of Postoperative Urinary Diversion on Surgical Outcomes of Hypospadias Repair: A Systematic Review and Meta-Analysis of Pediatric Literature
Source: Medicina (Kaunas). 2025 Sep 12;61(9):1659. doi: 10.3390/medicina61091659 (PMC12471891; doi:10.3390/medicina61091659)

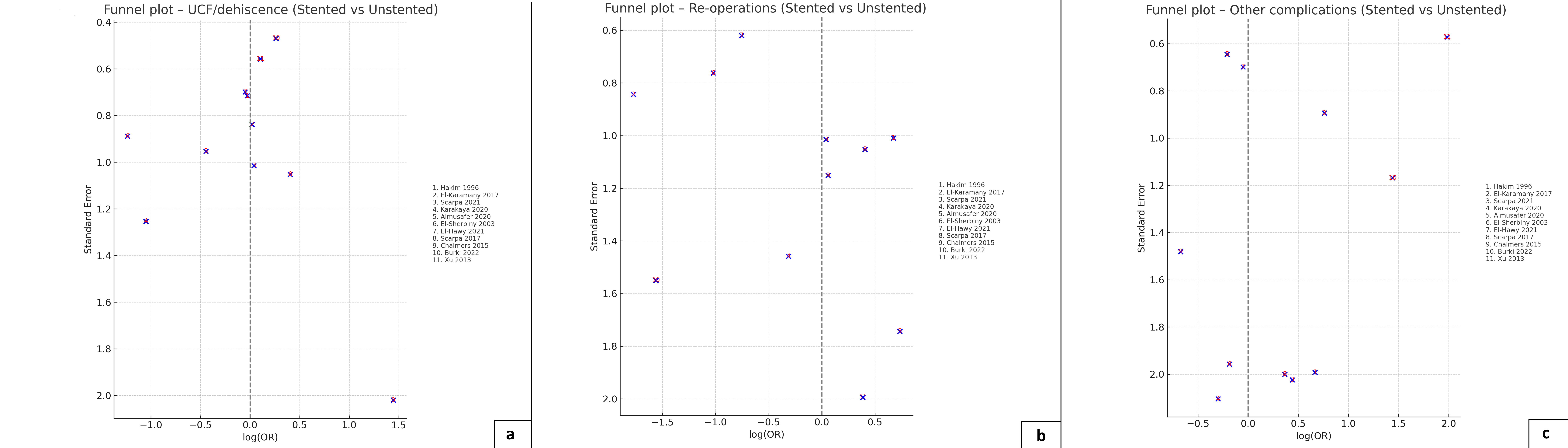

Supplement: Supplementary file 1 [file medicina-61-01659-s001.zip › Supplementary Figure S1.png]

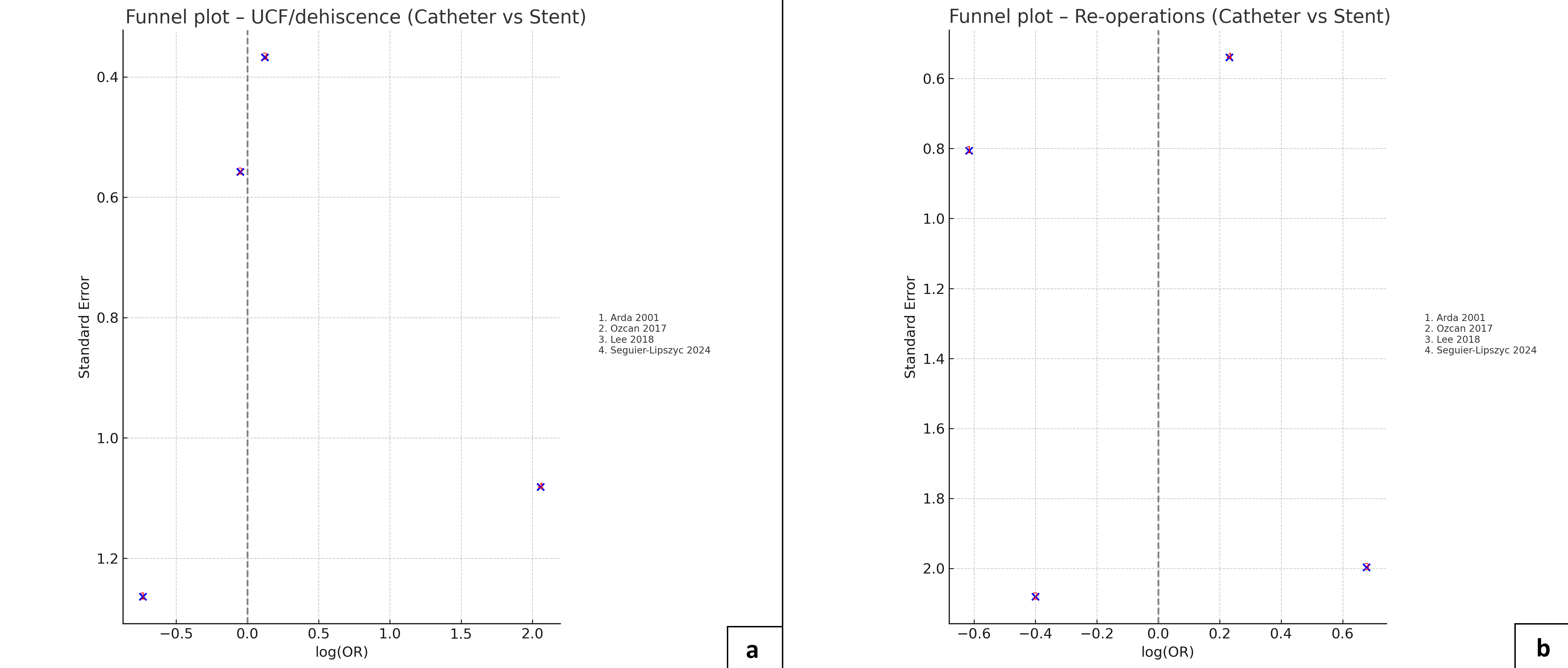

Supplement: Supplementary file 1 [file medicina-61-01659-s001.zip › Supplementary Figure S2.png]

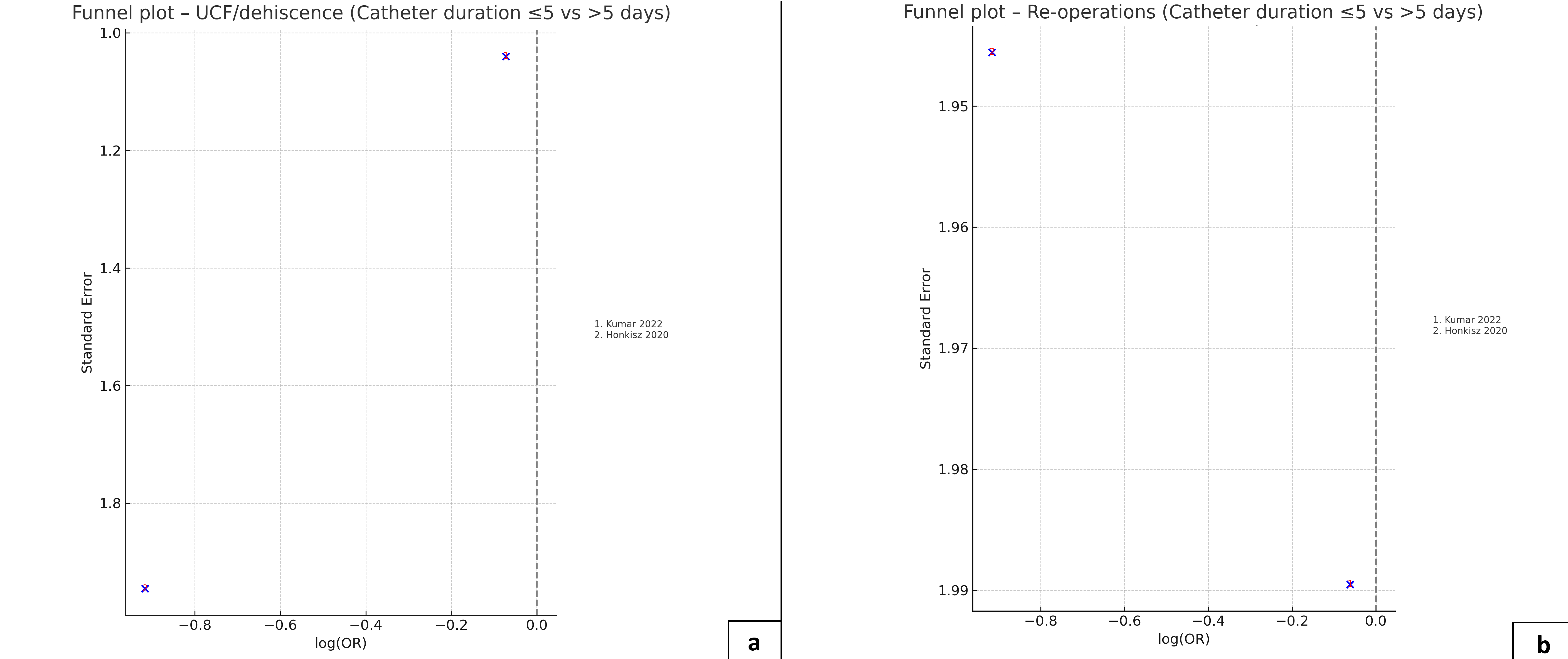

Supplement: Supplementary file 1 [file medicina-61-01659-s001.zip › Supplementary Figure S3.png]
